# Does osteotomizing the lower border of the mandible affect the lingual split pattern in a sagittal split ramus osteotomy?

**DOI:** 10.1186/s13005-023-00396-9

**Published:** 2023-11-07

**Authors:** Alah Dawood Al-Dawoody, Shehab Ahmed Hamad, Khurshid A. Kheder Khrwatany, Twana Hoshyar Saleem

**Affiliations:** 1 Al-Manara College for Medical Sciences, Mysan, Iraq; 2 Kurdistan Higher Council of Medical Specialties, Erbil, Iraq; 3 College of Dentistry, Hawler Medical University, Erbil, Iraq

**Keywords:** Bad split, Sagittal split, Inferior border cut, Lingual split, Mandible, Ramus

## Abstract

**Aim:**

The purpose of this study was to evaluate the effect of adding a fourth osteotomy at the lower border of the mandible on the lingual cortical fracture pattern in bilateral sagittal split ramus osteotomies.

**Patients and methods:**

The sample of the study consisted of 20 patients (12 male and 8 female, with a mean age of 26.79 ± 7.12 years) with mandibular deformities who needed bilateral sagittal split ramus osteotomy. One side underwent a traditional sagittal split ramus osteotomy, and the procedure was modified on the other side by adding a 1 cm horizontal osteotomy at the lower border of the mandible, just distal to the caudal end of the vertical buccal osteotomy cut. A 3D CBCT was used to identify the split pattern.

**Results:**

In the total sample, 40% of the lingual splits ran vertically toward the lower border of the mandible (LSS1), 20% of the splits passed horizontally to the posterior border of the mandible (LSS2), 32.5% of the splits took place along the inferior alveolar canal (LSS3), and 7.5% of the splits were unfavourable fractures (LSS4). On the inferior border osteotomy sides, the distribution of LSS1, LSS2, LSS3, and LSS4 was 10 (25%), 6 (15%), 4 (10%), and 0 (00), respectively. Their distribution on the sides without inferior border osteotomy was 6 (15%), 8 (20%), 13 (32.5%), and 3 (7.5%), respectively. Statistical analysis revealed a significant difference between the two groups (*p* < 0.05).

**Conclusion:**

Inferior border osteotomy tends to direct the lingual split fracture line toward the lower and posterior borders of the mandible and minimizes bad splits; however, further studies are needed to confirm our findings.

## Introduction

Bilateral sagittal split ramus osteotomy (BSSO) is a frequently performed orthognathic surgery to address a variety of jaw deformities, such as mandibular prognathism, retrognathism, and asymmetry [1]. The original BBSO of the mandibular ramus was initially described by Trauner and Obwegeser in 1957. In the original technique, a horizontal osteotomy was executed through the lingual cortex above the mandibular foramen. Subsequently, a second horizontal osteotomy was made on the buccal side, positioned lower than the lingual cut. These two horizontal osteotomies were connected by a third vertical osteotomy. The distal portion of the mandible, which contains the alveolar process and inferior alveolar canal, and the proximal segment, which includes the condyle and coronoid processes, are then separated into two segments [2].

Since its inception, numerous authors have explored various modifications aimed at improving bone contact between segments, reducing the risk of injury to the inferior alveolar nerve, decreasing bleeding complications, simplifying condylar positioning, and reducing the potential for relapse. Dal Pont [3] introduced an enhancement by extending and angling the lower horizontal cut further toward the buccal cortex of the mandibular body, with a vertical cut placed between the first and second molars. In addition to addressing conditions such as retrognathism and open bite, Dal Pont proposed a retromolar vertical osteotomy to minimize displacement of the proximal segment caused by the action of the pterygomasseteric sling elevator. To prevent the fracture from extending to the posterior border of the ramus, Hunsuck [4] recommended terminating the medial cortical osteotomy just posterior and superior to the mandibular foramen.

The Dal Pont approach was modified by Gallo et al. [5], particularly to address retrognathism. They proposed that the distal segment's vertical retromolar osteotomy should commence at the external oblique line and extend to the lower mandibular. The osteotomy trace is moved horizontally in the direction desired for mandibular advancement in order to define a step that is larger than the anticipated advance. The vertical osteotomy is then resumed in a more anterior position.

Epker [6] claimed that it was unnecessary to remove the pterygomasseteric sling from the ramus and that a full osteotomy of the inferior border of the lower jaw would lessen the risk of unintended fractures of the distal or proximal segments as well as injury to the inferior alveolar nerve. Wolford et al. [7] designed a vertical osteotomy from distal to the second molar, perpendicular to the inferior border of the mandible, with the cut extending through the lingual cortex and adding an osteotomy of the inferior border, stopping distal to the second molar, followed by a horizontal cut 8–10 mm below the alveolar bone crest. Verweij et al. [8] described an inclined vertical buccal bone cut that made an approximately 45° angle with the inferior border of the mandible. It commences from the external oblique ridge just distal to the second molar and extends toward the mandibular angle, ending close to the masseteric tuberosity.

To facilitate the surgeon’s ability to easily split the mandible, Wolford and Davis [9] incorporated a caudal (fourth) osteotomy cut along the lower border of the mandible. This cut extends from the mandibular angle to the lower end of the vertical buccal cortical osteotomy. Lower medial cortical osteotomies and a fourth horizontal osteotomy immediately above and parallel to the lower border were carried out by Mont’Alverne et al [10].

The most common complications associated with sagittal split osteotomies include severe bleeding, unfavourable fractures, significant oedema, infections, injury to the inferior alveolar nerve, and the potential for relapse. Among these, unfavourable fractures, injury to the inferior alveolar nerve, and relapse are the most critical issues, as they typically have a lasting impact on the patient’s long-term health [11]. In a comprehensive literature review, Chrcanovic and Freire-Maia [12] reported an overall incidence of 2.3% per BSSO, with the incidence varying between 0.21% and 22.72%. Bad splits have been observed in various locations during BSSO, including the coronoid process, condylar neck, lingual plate of the distal segment, and buccal plate of the proximal segment, with the latter type being the most frequently affected site of fracture [12].

Following BSSO, approximately 5–10% of patients may experience persistent injury to the inferior alveolar nerve [13, 14]. Neurosensory disturbance can lead to significant comorbidity, and neurosensory abnormalities like hyperaesthesia, paraesthesia, and dysaesthesia often exacerbate patients’ complaints [15]. Neurosensory disturbances can develop when there is contact with the nerve during surgery or when genioplasty is performed [16]. Since the inferior alveolar nerve is situated within the osteotomy area, the potential for nerve injury exists during BSSO surgery. Several risk factors for postsurgical neurosensory disturbance have been identified, including age, gender, nerve exposure, nerve manipulation, degree of mandibular mobility, surgical approach, and mandibular shape [17, 18].

The objective of this clinical study was to evaluate how the addition of a fourth osteotomy along the lower border of the mandible impacts the lingual split pattern and the occurrence of unfavourable fractures during a sagittal split ramus osteotomy.

## Patients and methods

### Study participants

The study sample consisted of individuals who underwent orthognathic surgery at a governmental hospital between February 1, 2013 and March 18, 2022. It included 20 patients who required treatment for conditions such as prognathism, retrognathism, or asymmetry and underwent BSSO. Patients with a history of prior mandibular trauma or any systematic disease were excluded from the study. Additionally, individuals older than 40 years were not considered for inclusion.

The 40 sagittal split osteotomies were divided into two groups using a split-mouth model. On one side, a conventional BSSO was performed, while on the other side, a lower border horizontal osteotomy was added. The study received approval from the Institutional Review Board, and adhered to the principles outlined in the Helsinki Declaration of Human Studies. Informed consent was obtained from all participating subjects.

### Operative procedure

All surgeries were carried out by the same surgeon (S.A.H) following the Hunsuck modification of the traditional Obwegeser/Dal Pont method. The surgery was conducted under general anaesthesia with nasotracheal intubation. The operative field was infiltrated with two anaesthetic cartridges of 2% lidocaine with 1:100,000 adrenaline. An incision was made in the buccal vestibule, near the second molar region, using a No. 15 blade. This incision was deepened to the bone to expose the anterior aspect of the lateral ramus and the posterior body, reaching the inferior border.

Following the incision, the temporalis muscle tendon was stripped, exposing the anterior superior aspect of the ramus up to the coronoid process. A medial soft tissue dissection was performed to reveal the mandibular foramen. Retraction of the soft tissues was achieved using specific instruments, including a channel retractor positioned superior to the neurovascular bundle, a fork retractor on the coronoid process, and an inferior border retractor placed anterior to the gonial angle.

The lingual cortical osteotomy was executed using a long fissure bur, and it terminated just posterior and superior to the mandibular foramen. The buccal vertical osteotomy was conducted with a fissure bur in the area between the first and second molars, extending from the external oblique ridge to the lower border of the mandible. These osteotomies were connected by a third osteotomy along the external oblique ridge.

**Fig. 1:**
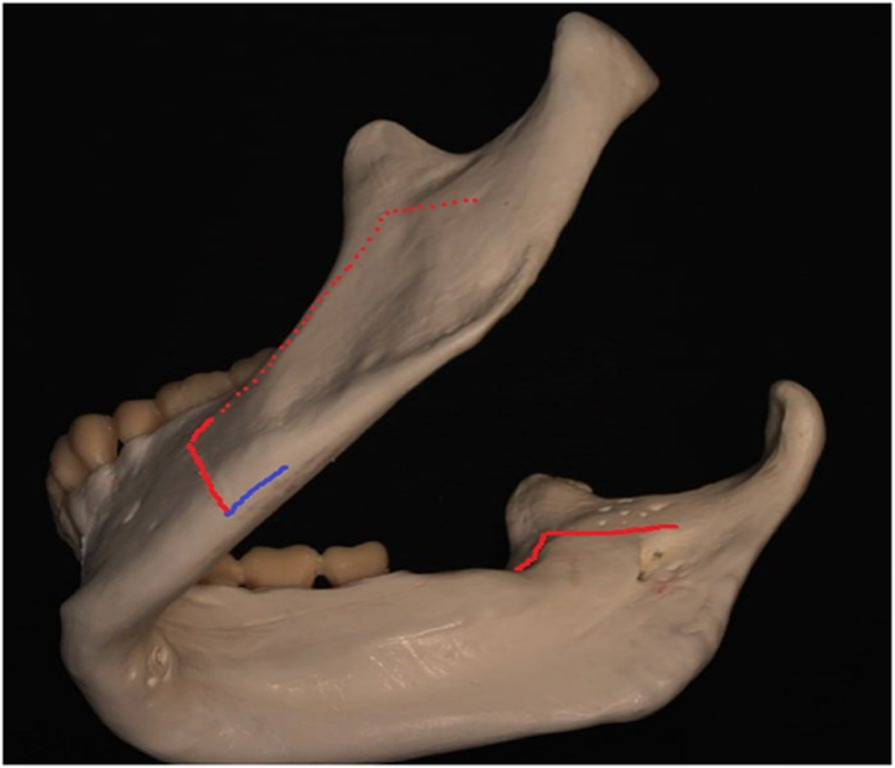
Lower border osteotomy (blue colour)

On the experimental side, an additional fourth osteotomy, approximately 10 mm in length, was carried out along the inferior border of the mandible (Fig. 1) using a right-angle piezosurgery saw tip. The osteotomy was then completed using a chisel, mallet, and Smith’s spreader. Following the osteotomy procedures, the condyle positions were verified, and fixation was achieved using a 4-hole straight miniplate. Closure of the wound was accomplished using absorbable sutures. All patients were placed on a loose intermaxillary fixation for a duration of seven days.

After the surgery, all patients were administered intravenous diclofenac (75 mg) and paracetamol (1000 mg). Additionally, they were given 1.2 g of intravenous amoxicillin-clavulanic acid 30 min prior to surgery, followed by 625 mg tablets every 8 h for the first five days following the procedure. Allergic patients received clindamycin at a dose of 600 mg intravenously three times daily for the first two days and then switched to 300 mg tablets taken orally three times daily for the subsequent five days.

Each patient received comprehensive instructions on postoperative oral hygiene maintenance, including guidance on using a soft toothbrush and an antiseptic mouthwash (chlorhexidine gluconate 0.1%). During their hospital stay, the patients underwent daily examinations. After being discharged, appointments were scheduled on a weekly basis for six weeks to monitor healing progress, assess any infection symptoms, evaluate oral hygiene, and check occlusion.

### Data acquisition

Using cone beam computed tomography (CBCT) (PLANMECA PROMAX 3D-MID, Finland), scans were conducted on the 7th day postoperatively to assess the fracture pattern of the lingual split and to identify the presence of unfavourable splits.

**Fig. 2:**
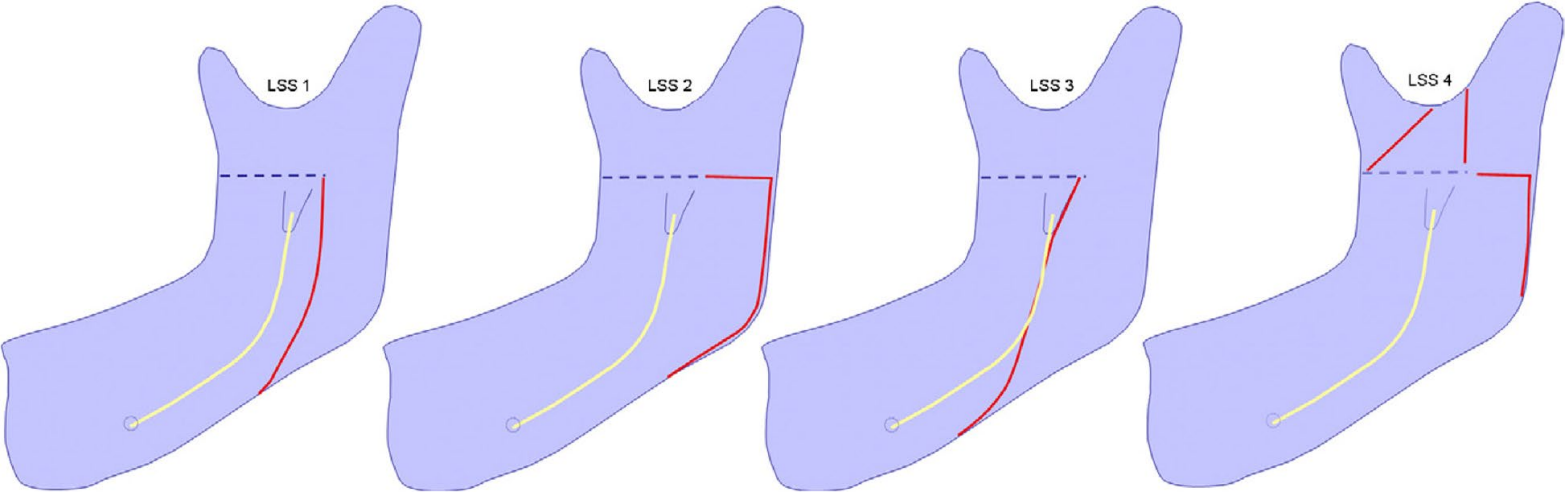
Lingual split pattern in BSSO (Plooij JM et al [19])

The fracture patterns were categorized using the Lingual Split Scale (LSS) as described by Plooij et al. [19] (Fig. 2). The categorizations were as follows:

- LSS1: Vertical fracture line extending to the inferior border of the mandible, following Hunsuck’s description.
- LSS2: Horizontal fracture line extending to the posterior border of the ramus, as originally described by Obwegeser/Dal Pont.
- LSS3: Fracture line passing through the mandibular canal to reach the inferior border of the mandible.
- LSS4: Other patterns, such as a buccal plate fracture or unfavourable split.

### Statistical analysis

Statistical analysis was conducted using the IBM SPSS version 28 software program for Windows (IBM Corp., Armonk, NY, USA). A Z-test for proportion was employed to assess the statistical difference in the occurrence of bad splits between cases of BSSO with inferior border osteotomy and those without. Fisher’s exact test was used to determine the statistical significance of the lingual fracture pattern’s frequency between the two groups. The level of significance was set at a *p*-value of ≤ 0.05.

## Results

Out of the 20 patients who met the inclusion criteria, 12 (60%) were male and 8 (40%) were female. Their mean age was 26.79 ± 7.12 years, with an age range of 18–40 years). Among these patients, 14 (70%) fell within the age group of 20–30 years, while 6 (30%) were in the age group of 31–40 years.

Regarding the surgical procedures performed, 8 cases (40%) of BSSO were performed alone; 3(15%) cases were combined with genioplasty; 6 (30%) cases were combined with Le Fort I osteotomy; and 3 (15%) cases were combined with both Le Fort I osteotomy and genioplasty. Setback was performed in 8 (40%) patients, advancement in 10 (50%) patients, and rotation in 2 (10%) patients (Table 1).

**Table 1:** Demographic characteristics of the patients

Variables	No. (%)
Age:	
20–30 yrs	14 (70)
31–40 yrs	6 (30)
Sex:	
Male	12 (60)
Female	8 (40)
Surgery type:	
BSSO alone	8 (40)
BSSO + genioplasty	3 (15)
BSSO + Le Fort I osteotomy	6 (30)
BSSO + Le Fort I osteotomy + genioplasty	3 (15)
Direction of mandibular movement:	
Setback	8 (40)
Advancement	10 (50)
Rotation	2 (10)

No instances of bad split were observed in the sites where additional inferior border osteotomies were performed. However, 3 (15%) cases of bad split occurred on the other side, where no inferior border osteotomies were carried out. It’s worth noting that the difference was not statistically significant (*P* = 0.07) (Table 2). Specifically, one case involved a fractured lingual plate of the distal segment, while two cases exhibited a fracture of the buccal plate of the proximal segment.

**Table 2:** Distribution of bad splits in the two groups

	Bad split No. (%)	No bad split No. (%)	Total No. (%)	*P* Value^*^
BSSO with inferior border osteotomy	0 (00)	20 (100)	20 (50)	0.07
BSSO without inferior border osteotomy	3 (15)	17 (85)	20 (50)	
Total, No. (%)	3 (7.5)	37 (92.5)	40 (100)	

^*^Z test of proportions

There was, however, a statistically significant difference in the distribution of the lingual cortical split patterns between the two groups (*P* = 0.04). In the overall sample, the distribution of the patterns LSS1, LSS2, LSS3, and LSS4 was as follows: 16 (40%), 8 (20%), 13 (32.5%), and 3 (7.5%), respectively. On the sides with inferior border osteotomies, the distribution was 10 (25%), 6 (15%), 4 (10%), and 0 (0%), respectively. In contrast, on the sides without inferior border osteotomies, the distribution was 6 (15%), 8 (20%), 13 (32.5%), and 3 (7.5%), respectively (Table 3).

**Table 3:** Distribution of lingual cortical fracture patterns in the two groups

	Lingual cortical fracture pattern^a^, No. (%)					*P* Value
	LSS1	LSS2	LSS3	LSS4	Total	
BSSO with inferior border osteotomy	10 (25)	6 (15)	4 (10)	0 (00)	20 (50)	0.04
BSSO without inferior border osteotomy	6 (15)	2 (5)	9 (22.5)	3 (7.5)	20 (50)	
Total, No. (%)	16 (40)	8 (20)	13 (32.5)	3 (7.5)	40 (100)	

^a^According to the lingual split score by Plooij et al. [19], Fisher’s exact test

## Discussion

The aim of this study was to assess the impact of incorporating a fourth osteotomy along the lower border of the mandible on the lingual split pattern during BSSO, using 3D CBCT. The results of this study showed that the original Obewgeser/Dal Pont complete lingual horizontal fracture extending toward the posterior border of the mandible, as well as the Hunsuck fracture pattern that runs vertically toward the lower border of the mandible, behind the mandibular foramen, are favourably affected by the addition of a fourth osteotomy cut at the lower border of the mandible. It is noteworthy that no undesirable fracture patterns were observed on the sides with a horizontal lower border osteotomy, in contrast to three cases of unfavourable fractures patterns on the opposite side without an inferior border osteotomy.

Sagittal split ramus osteotomy remains the established standard for correcting mandibular skeletal deformities. Since its introduction by Obwegeser in the mid-50 s, numerous modifications have been introduced to facilitate the splitting process, reduce complications, expedite bone healing, and prevent relapse. The addition of a fourth osteotomy cut along the lower border serves to further weaken the mandibular body and streamline the splitting process. Access to the lower border can be challenging and may necessitate extensive soft tissue manipulation and potential trauma; however, the utilization of a piezotome can mitigate the level of trauma involved.

The lingual split pattern is inherently concealed and not clinically visible. However, with the advent of CBCT technology, predicting and visualizing the fracture pattern has become more accessible. In a study focused on BSSO for mandibular advancement involving 40 patients with mandibular hypoplasia, Plooij et al. [19] introduced a new scale to categorize the path of the lingual cortical fracture line. Their findings revealed that 51.25% of splits followed Hunsuck’s description (LSS1), 13.75% of fractures extended horizontally to the posterior border (LSS2), 32.5% of lingual fractures occurred along the inferior alveolar canal (LSS3), and 2.5% were buccal or categorized as other unfavourable fracture types (LSS4).

In the current study, the distribution of lingual split patterns was as follows: LSS1 occurred in 40%, LSS2 in 20%, LSS3 in 32.5%, and LSS4 in 7.5%. The inclusion of a lower border osteotomy cut has advantages in terms of the lingual fracture pattern. Specifically, there was a tendency toward LSS1 and LSS2 fracture patterns compared to the conventional technique without an inferior border cut, which showed a propensity for LSS3 fracture patterns. Unfavourable splits (LSS4) were exclusively observed in the traditional technique without the inferior border cut.

Notably, the LSS1 and LSS2 split patterns are located away from the mandibular canal, potentially reducing the risk of inferior alveolar nerve damage. These fracture patterns also increase the bone contact surface area, which proves advantageous in cases involving mandibular advancement. Our findings align with previous studies conducted on cadaveric animals [20, 21] and a cadaveric human study [22]. However, our results diverge from the clinical studies of Houppermans et al. [23] and Mohlhenrich et al. [24], who reported no significant association between the inferior border cut and the lingual split pattern.

The variation in outcomes among different studies can be attributed to several factors, including differences in anatomy between human and animal models, the expertise of the surgeon, the sample size, and the influence of factors like ethnicity, age, sex, the presence or absence of third molars, the degree of mandibular divergence, and variations in the size and shape of the mandible. The size and shape of the mandible can vary significantly among individuals due to a complex interplay of genetic factors [25], environmental infleunces [26], sex-related variations [27], and geographic and ethnic differences [28]. Additionally, variations in the tools used for osteotomy and splitting procedures may contribute to this diversity.

Dal Pont proposed a pattern that enhances the common surface area between the split bone segments, thereby promoting bone integrity. This modification allows for further displacement of the distal segment and has been shown to be effective [29]. The Hunsuck and Epker modification, on the other hand, involves an incomplete lingual osteotomy that terminates just behind the lingula and inferior alveolar canal. This modification is notably easier compared to the conventional lingual osteotomy, which traditionally extends to the posterior border of the ramus and may occasionally result in unfavourable split fractures [30].

The occurrence of unfavorable splits is genuinely possible with both Hunsuck and Dal Pont modifications. Therefore, selecting a technique that carries the least risk of encountering such complications can undoubtedly lead to a more satisfactory surgical outcome. A study conducted by Zamiri et al. [31] involved an evaluation and comparison of fracture patterns within the medial cortex resulting from both medial long-cut and medial short-cut techniques during BSSO. In their investigation, they identified three distinct fracture patterns but found no significant correlation between the type of medial cut and the resulting fracture pattern. Consequently, it was concluded that the length of the medial cut does not significantly influence the occurrence of unfavorable split fractures. Zeynalzadeh et al. [32] reported that when using the Hunsuck approach, both osteotomies and splitting procedure require significantly less time, and there are also fewer instances of unfavorable fractures compared to when employing the Dal Pont osteotomy technique.

Cortical bone thickness has also been implicated as a risk factor for bad splits. In the study of Arabi et al. [33], they observed that the average buccolingual thickness of the retromandibular area measured 14.98 mm in the group of patients who experienced poor split outcomes. This measurement significantly differed from the average thickness observed in their control group, which stood at 11.21 mm. Additionally, the buccolingual thickness of the ramus at the lingula level was found to be associated with unfavorable split results. The occurrence of unfavorable intraoperative splitting has been linked to a limited gap between the inferior alveolar nerve canal and the buccal cortex, as well as a reduction in the thickness of both the buccal cancelous bone and the overall cancelous bone along the path of the splitting [34].

The height of the lingual osteotomy and the amount of cancelous bone between the ramus cortices may also be contributing factors to bad splits. Numerous studies have indicated that when the lingula is positioned high on the mandibular ramus, the medial horizontal osteotomy needs to be performed at a higher level on the mandibular ramus, specifically in a thin area where there is minimal or no cancelous bone present [35]. In skeletal class III malocclusions, the lingula tends to be situated higher compared to class I and class II malocclusions, and the ramus often displays limited marrow. Consequently, class III malocclusions are commonly associated with the highest risk of experiencing unfavourable fractures [36].

This study does have certain limitations, including the challenge of controlling the inferior border osteotomy cut due to limited access. Furthermore, despite employing a split-mouth design, it;s important to acknowledge the presence of anatomical variations between the two sides, which may impact the results.

## Conclusion

The modification of the traditional sagittal split ramus osteotomy by adding a fourth osteotomy cut at the lower border of the mandible favours the original Obewgeser/Dal Pont complete lingual horizontal fracture extending toward the posterior border of the mandible, as well as the Hunsuck fracture pattern that runs vertically toward the lower border of the mandible, behind the mandibular foramen. It is recommended to carry out additional studies with a larger sample size to gain a more comprehensive understanding of the relationship between study variables.

## Data Availability

Data are available on request by contacting the corresponding author.
